# Polyphenols and Pharmacological Screening of a *Monarda fistulosa* L. dry Extract Based on a Hydrodistilled Residue By-Product

**DOI:** 10.3389/fphar.2021.563436

**Published:** 2021-04-29

**Authors:** Mariia Shanaida, Nataliia Hudz, Izabela Jasicka-Misiak, Piotr P. Wieczorek

**Affiliations:** ^1^ Department of Pharmacognosy and Medical Botany, I. Horbachevsky Ternopil National Medical University, Ternopil, Ukraine; ^2^ Department of Drug Technology and Biopharmaceutics, Danylo Halytsky Lviv National Medical University, Lviv, Ukraine; ^3^ Faculty of Chemistry, University of Opole, Opole, Poland

**Keywords:** wild bergamot, herb, postdistillation waste, phenolic compounds, safety, anti-inflammatory activity, antiradical activity, analgesic activity

## Abstract

This study aimed to determine the composition and content of polyphenols in the dry extract obtained from the hydrodistilled residue by-product of the wild bergamot (*Monarda fistulosa* L., *Lamiaceae* Martinov family) herb (MFDE) and to evaluate its safety and pharmacological properties. The total phenolic content (TPC) in the MFDE was 120.64 mg GAE/g. The high-performance liquid chromatography (HPLC) analysis showed the presence of a plethora of phenolic compounds, including hydroxycinnamic acids and flavone derivatives in the MFDE, with rosmarinic acid and luteolin-7-*O*-glucoside being the main components. With an IC_50_ value of 0.285 mg/mL, it was found to be a strong DPPH radical scavenger. The acute toxicity study results indicate that the oral administration of MFDE to rats at the doses of 500–5,000 mg/kg did not produce any side effects or death in animals which indicates its safety. The results of the *in vivo* assay showed that the MFDE dose-dependently inhibited paw oedema and significantly reduced the number of writings in mice induced by the acetic acid injection suggesting its potent anti-inflammatory and analgesic activities, respectively. The conducted studies revealed that *M. fistulosa* hydrodistilled residue by-product could be regarded as a new natural source of polyphenols with valuable pharmacological properties.

## Introduction

Genus *Monarda* L. (*Nepetoideae* Burnett. of the *Lamiaceae* family) includes several species of herbaceous plants native to North America (Native plants, 2001)*.* Nowadays, *Monarda* species are widely cultivated as ornamental and medicinal plants for local use, but they are not included in any Pharmacopoeia (American Herbal Pharmacopoeia, 2011; European Pharmacopoeia, 2016). The value of *Monarda* species is mainly based on their volatile constituents and ornamental features (Vysochina, 2020). It was found that only 49 original studies and reviews were retrieved from PubMed up to November 2020 about the chemical composition and biological activities of species from the *Monarda* genus (data obtained via searching the word “*Monarda*”). Scientifically confirmed pharmacological activities of *Monarda* plants were primarily revealed by the researchers due to the composition of essential oils isolated from the aerial parts of *M. fistulosa* L.*, M. didyma* L.*, M. citriodora* Cerv. ex Lag., and *M. punctata* L. (Fraternale et al., 2006; Zhilyakova et al., 2009; Geffre et al., 2011; Tabanca et al., 2013; Thompson et al., 2013; Li et al., 2014; Mattarelli et al., 2017; Shanaida et al., 2018b; Deepika et al., 2020; Ghosh et al., 2020; Marchioni et al., 2020).

The other groups of biologically active compounds of *Monarda* genus representatives such as flavonoids or hydroxycinnamic acids were studied less. Some of the scientific studies about the polyphenols composition of these species were published not only in the peer-reviewed journals (Banach and Olechnowicz-Stepien, 1986; Davies and Mazza, 1992; Savickiene et al., 2002; Krasyuk and Pupyrkina, 2016; Shanaida et al., 2018a; Shanaida et al., 2020; Vysochina, 2020). Flavonoids such as rutin, hyperoside, quercetin, quercitrin, and luteolin were found in the leaves and flowers of *M. didyma* grown in Lithuania by the HPLC method (Savickiene et al., 2002). Rosmarinic acid and flavonoids hyperoside, rutin, naringin, and naringenin were identified in the *M. didyma* herb by the high-performance thin layer chromatography (HPTLC) (HPTLC method for identification of medicinal plants (method collection), 2012). Davies and Mazza (1992) found that anthocyanin pelargonidin 3,5-diglucoside accounted for 17% of the total flavonoid content in the petals of *M. fistulosa* flowers. Among the other detected flavonoids were apigenin-7-*O*-glucosides, dihydroxyflavone 8-*C*-glucoside, and 5-hydroxyflavone (Davies and Mazza, 1992). Six flavonoid glucosides were isolated from the *M. pectinata* aerial part, i.e., acacetin-7-rutinoside, isosacuranetine-7-rutinoside, and luteolin-7-glucoside (Banach and Olechnowicz-Stepien, 1986). It was found that *Monarda* species introduced in the Republic of Bashkortostan (Russia) accumulated the total content of flavonoids calculated as luteolin equivalent at the levels of 1.57% in *M. fistulosa*, 1.63% in *M. didyma*, 1.61% in *M. citriodora*, 1.52% in *M. hybrida* Wender., and 0.91% in *M. russelliana* Nutt. (Krasyuk and Pupyrkina, 2016). Rosmarinic acid was found to be the main component of aqueous and methanol extracts obtained from the *M. fistulosa* herb grown in Ukraine (Shanaida et al., 2018a; Shanaida et al., 2020). The preventive and healing properties of polyphenols are mostly related to their antioxidant effects which could be useful in the treatment of inflammation, pain, cardiovascular diseases, neurodegenerative disorders, and cancer (Pudziuvelyte et al., 2020). Many chronic diseases of human and animal bodies lead to releasing reactive oxygen or nitrogen species, causing cell injury mainly through their oxidative degradation (Libby, 2007). As a result, inflammation is developing, nociceptors are sensitized, and it becomes painful (Luo et al., 2020).

Wild bergamot or bee balm (*M. fistulosa*) is a perennial herbaceous plant 60–90 cm tall, with erect branches and deltoid-lanceolate opposite leaves; its violet-pink corolla is strongly bilabiate; flowers are collected in the compact clusters 4-5 cm long at the tops of stems (Wagner, 2006; Thompson et al., 2013). This plant is easily cultivated, and as a consequence, it is widespread on different continents in a temperate climate as a garden ornamental, honey source, and medicinal plant (Krasyuk and Pupyrkina, 2016; Vysochina, 2020). *M. fistulosa* has been successfully introduced to the European countries. Research institutions are conducting the acclimatization and breeding of new cultivars of this species (Vysochina, 2020). Historically, among the *Monarda* representatives, *M. fistulosa* has been the most widely used effective local medicine across American Indian cultures in North America (Native plants, 2001)*.* The aerial parts of this plant were used by many Native Americans to cure cold and flu, for the treatment of the oral cavity infections (gingivitis or dental caries), as a strong antiseptic for skin wounds, as an active diaphoretic, to relief headache and the flatulent colic, and in waterbaths for babies (Native plants, 2001; Vysochina, 2020; Wagner, 2006). The Cherokee used a warm poultice of bee balm to relieve headache (Wagner, 2006). Flambeau Ojibwe dried the whole aerial part of this plant and boiled it in a suitable vessel to obtain the essential oil for treatment of bronchial and catarrh conditions through inhalation (Wagner, 2006). Later, it was found that *M. fistulosa* essential oil is the natural source of thymol and *p*-cymene with strong antiseptic properties (Zhilyakova et al., 2009; Mattarelli et al., 2017).

Postdistillation wastes are obtained after the extraction of essential oils from the raw material of the plants belonging to the *Lamiaceae, Asteraceae, Apiaceae*, and *Pinaceae* families. It seems that using postdistillation wastes is quite a perspective in terms of the extraction of new biologically active substances possessing valuable pharmacological properties. As the amount of essential oil in the aerial parts of the Lamiaceae family representatives is not high (up to 3%), the majority of a plant raw material remains unused after the hydrodistillation (Gavarić et al., 2015). This scientific and practical field of using hydrodistilled residue by-products has been expanding rapidly for the species of this family for the last decade (Gavarić et al., 2015; Pogačar et al., 2016; Vovk et al., 2016; Baranauskienė et al., 2019; Mahmoudi et al., 2020; Shanaida, 2020). Nevertheless, HPLC assay revealed the high contents of such valuable polyphenols as rosmarinic acid (52.36–105.8 mg/g) and rutin (11.01–87.11 mg/g) in the dried extract developed from the postdistillation waste material of *Thymus vulgaris* L. (Gavarić et al., 2015). In another study (Pogačar et al., 2016), it was revealed that the extract obtained from the *Thymus vulgaris* post-hydrodistillation residue was rich in such bioactive phytochemicals as rosmarinic acid and flavone glucuronides. This extract effectively reduced the adhesion of *Campylobacter jejuni* to abiotic surfaces at low concentrations (0.2–12.5 μg/mL) (Pogačar et al., 2016). Hydrodistilled residue by-products from *Ocimum basilicum* L. leaves were also considered as a valuable source of rosmarinic acid and other polyphenols with noticeable antimicrobial, repellent, and antioxidant effects (Mahmoudi et al., 2020). Solid residues of *Lavandula* × *intermedia* and *Thymus mastichina* L. after the isolation of the essential oil were found as a source of polyphenols with antioxidant and metal chelating activities (Sánchez-Vioque et al., 2013). To the best of our knowledge, there are no studies related to hydrodistilled residue by-product of *M. fistulosa* aerial part.

The aim of this study was to characterize the phenolic profile in the dry extract developed from the hydrodistilled residue by-product of *M. fistulosa* herb (MFDE), to determine its safety, free radical scavenging, anti-inflammatory, and analgesic activities.

## Materials and Methods

### Plant Material

The herb of *M. fistulosa* was harvested during the flowering stage from the experimental plots in Ternopil Region (Ukraine, 49.5535°N, 25.5948°E), then dried in the shadow at a temperature of 25–35°C, and ground. The voucher specimens of the plant have been deposited in the herbarium of Pharmacognosy and Medical Botany Department of I. Horbachevsky Ternopil National Medical University.

### Preparation of the MFDE

The MFDE was obtained from the postdistillation waste of *M. fistulosa* herb in two stages (Shanaida, 2020): by aqueous extraction in the hydrodistillation process and by using 50% ethanol at the next stage as it was described for *Salvia officinalis* L*.* leaves by Vovk et al. (2016). 120.0 g of ground herb of *M. fistulosa* was used for obtaining the MFDE. The raw plant material was divided into four portions and subjected to hydrodistillation for 2 h (the ratio of the raw material to purified water was 1:15) (European Pharmacopoeia, 2016). After the separation of essential oil (1.87%), the aqueous extract was cooled, then filtered through a paper filter, and maintained for 24 h in a refrigerator (Shanaida, 2020). On the next stage, the decanted solution was evaporated in a vacuum rotary evaporator to 1/10 of an initial volume. Then, 1.2 L of 50% ethanol was added to the waste, and it was extracted using a boiling water heater for 40 min. After cooling, the filtrate was put in the refrigerator (for 24 h) to sediment ballast, then decanted, and evaporated to 1/10 of an initial volume. The obtained concentrated extracts were combined and dried in a vacuum spray dryer to obtain the MFDE**.**

### Chemicals

Folin–Ciocalteu’s reagent, methanol, ethyl acetate, formic acid, acetic acid, gallic acid, Na_2_CO_3_, and AlCl_3_ were purchased from POCH S.A. (Gliwice, Poland). Standards of apigenin, luteolin, rutin, rosmarinic acid, caffeic acid, and chlorogenic acid for HPTLC, 2,2-diphenyl-1-picrylhydrazyl (DPPH), Trolox, carrageenan, and Tween-80 were purchased from Sigma-Aldrich (Poland). HPTLC silica gel 60 F_254_ plates, trifluoroacetic acid, acetonitrile, and standards for HPLC (chlorogenic acid, neochlorogenic acid, rosmarinic acid, caffeic acid, luteolin-7-*O*-glucoside, apigenin-7-*O*-glucoside, acacetin-7-*O*-glucoside, luteolin, and apigenin) were purchased from Merck (Germany). The tablets of “Diclophenac” (produced by chemical and pharmaceutical factory “Chervona zirka”, Kharkiv, Ukraine) and “Metamizole sodium (Analgin)” (produced by pharmaceutical factory “Darnitsa”, Kyiv, Ukraine) were purchased from the pharmacies in Ukraine. All the reagents and solvents were of analytical grade.

### Total Phenolic Content

The TPC was measured according to the Folin–Ciocalteu method (Singleton and Rossi, 1965) with a Hitachi UV/VIS spectrophotometer and calculated as gallic acid equivalents (mg GAE/g extract). To plot the calibration curve, gallic acid was dissolved in purified water for obtaining the solutions in the range of 0.02–0.14 mg/mL (Shanaida et al., 2018a). Briefly, 0.1 mL of the aqueous solution of MFDE (concentration 0.5 mg/mL) was mixed with 1.5 mL of purified water, 0.1 mL of undiluted Folin-Ciocalteu reagent, and 0.3 mL of 20% Na_2_CO_3._ The obtained mixtures were incubated for 2 h in darkness at room temperature; the absorbance was measured with the spectrophotometer Hitachi U-2810 UV/VIS at 760 nm.

### Chromatographic Analyses

The HPTLC analysis of phenolic compounds was carried out using a CAMAG analytical system (Switzerland) (Stanek et al., 2019; Shanaida et al., 2020). The MFDE was dissolved in methanol (1.0 mg/mL) and filtered through a 0.45 μm Millipore filter to obtain a test solution. A standard solution consisted of the available reference standards dissolved in methanol (0.25 mg/mL). The test and standards solutions in a volume of 5 μL were applied to chromatographic plates (20 cm × 10 cm) using an automatic application device. The mobile phase consisted of ethyl acetate: formic acid: water (15:1:1). The detection of polyphenols spots was based on their natural fluorescence after the postchromatographic derivatization by 1% AlCl_3_ solution at 254 and 366 nm.

The HPLC analysis of polyphenols was performed using the Shimadzu HPLC-DAD system using the Phenomenex Luna C18 column (250 × 4.6 mm, 5 µm particle size) at 35°C. The UV absorption spectra of the available reference standards and the test samples were recorded in the range of 190–400 nm. Gradient elution conducted by mixing the mobile phases A (0.1% trifluoroacetic acid in water) and B (0.1% trifluoroacetic acid in acetonitrile) at a flow rate of 1.0 mL/min according to Shanaida et al. (2018a) (Table 1). The concentration ranges of reference standards for the calibration curve were 10.0–1000.0 μg/mL for rosmarinic acid and luteolin-7-*O*-glucoside and 1.0–100.0 μg/mL for other components; *R* ^2^ was not less than 0.99 for all measurements.

**TABLE 1:** Gradient of the mobile phases in HPLC analysis (Shanaida et al., 2018a).

Time (min) after injection of a sample	Mobile phase А (Vol, %)	Mobile phase B (Vol, %)
0–5	95	5
5–35	95 → 75	5 → 25
35–40	75	25
40–60	75 → 50	25 → 50
60–65	50 → 20	50 → 80
65–70	20	80
70–85	95	5

### DPPH Free Radical Scavenging Activity

The inhibition of DPPH radicals by the MFDE was studied using the Hitachi U-2810 UV/VIS spectrophotometer according to the method of Gougoulias and Mashev (2015). The MFDE and DPPH reagent were dissolved in methanol before the analysis. 0.1 mL of each test sample in various concentrations (0.1–1.0 mg/mL) was mixed in the flask with 1.9 mL of DPPH solution in methanol (25 μg/mL), and this mixture was incubated for 30 min in darkness at room temperature. The absorbance was measured at a wavelength of 517 nm. The concentration of the antioxidants presented in the test solution required to scavenge 50% of DPPH was expressed as an IC_50_ value; *R* ^2^ = 0.9906. Trolox was used as a standard antioxidant.

### Acute Toxicity, Anti-Inflammatory, and Antinociceptive Activities

All the *in vivo* experiments were conducted according to “European Convention for the Protection of Vertebrate Animals (1986)”.

**Animals:** mice (20–25 g) and albino rats (180–220 g) were used for the studies of acute toxicity and pharmacological activities of the MFDE. The animals were kept in plastic cages under the constant conditions (22 ± 2°C); they had free access to water and were fed *ad libitum*. After dividing into groups (*n* = 6–10), the animals fasted 12 h before the experiment.

**Acute Toxicity Study:** rats of both sexes in a ratio of 1:1 were orally given MFDE (500, 1,500, and 5,000 mg/kg bodyweight) once a day (*Doklinichni doslidzhennia likarskykh zasobiv (metodychni rekomendacii)*, (2001). Different doses of the MFDE were dissolved in the vehicle (1% solution of Tween-80 in distilled water) and put into the stomach of rats through a tube. The animals were observed daily up to 14-days following the treatment. The fatality, general behavior, breath depth and rhythm, salivation, feed and water consumption, the character of excrements, condition of wool, mucous membranes, and dynamics of bodyweight before and during the experiment were analyzed daily.

**Anti-Inflammatory Effect:** the hind paw oedema of rats was produced by carrageenan (*Doklinichni doslidzhennia likarskykh zasobiv (metodychni rekomendacii)*, (2001); Wang et al., 2012). Animals were divided into five groups (*n* = 6 animals/group). Each animal was injected with 0.1 mL of 1% carrageenan suspension into the right plantar aponeurosis for inducing the paw oedema. The animals were orally pretreated with the MFDE, diclofenac, or vehicle 60 min before the carrageenan injection. The rats in the control group were given the vehicle (1% solution of Tween-80 in distilled water), animals of 2–4 groups were pretreated with the MFDE in doses of 50, 100, or 200 mg/kg (experimental groups), and the 5th group was given the reference analgesic drug “Diclofenac” (8 mg/kg).

The paw volume oedema was measured plethysmometrically before and 1, 3, and 6 h after the injection of carrageenan. Anti-inflammatory activity (AIA) was expressed as the percentage reduction in oedema in the treated rats by comparison with the controls according to the following formula:

$$\%AIA=\left[\left(d_{control}-d_{treated}\right)/d_{control}\right]\times 100,$$

where *d*_treated_ is the difference in paw volume in the treated group, and *d*_control_ is the difference in paw volume in the control group.

**Antinociceptive Activity:** acetic acid induced stretching of the hind limbs and writhing of mice were provoked (*Doklinichni doslidzhennia likarskykh zasobiv (metodychni rekomendacii)*, (2001); Magne et al., 2016). The mice were divided into five groups (*n* = 6 animals/group). The animals of the experimental groups were orally pretreated with the MFDE (50, 100, or 200 mg/kg), and the reference group was given “Metamizole sodium” (55 mg/kg) in 60 min before the intraperitoneal injection of 0.6% solution of acetic acid. The animals of the control group were pretreated before the intraperitoneal injection of acetic acid with the vehicle (1% solution of Tween-80 in distilled water). The mice were individually placed into the chambers. The number of stretching the hind limbs and writhing was counted for 20 min.

The percentage of analgesic activity (AA) was expressed as percentage decreasing in the number of “acetic acid cramps” in the treated animals concerning the controls as follows:

%AA=[(control mean-treated mean)/control> mean]×100

### Statistical Analysis

Statistical analyzes were performed using the Statistica software, version 13.1 (StatSoft). The HPLC analysis and the evaluation of TPC and DPPH free radical scavenging activity were carried out in triplicate. The acute toxicity study and anti-inflammatory and antinociceptive activities were carried out at a minimum of *n* = 6. The data were expressed as mean ± standard error of the mean (SEM). The results were analyzed using ANOVA one-way analysis of variance coupled with Dunnett’s test.

## Results and Discussion

### Phytochemical Evaluation of MFDE

By using aqueous extraction in the hydrodistillation process and 50% ethanol at the next stage, a 23.17% MFDE yield was obtained. The TPC content was found to be 120.64 ± 2.65 mg GAE/g. This value was higher than those observed for other *Lamiaceae* species. Thus, Vlase et al. (2014) revealed that the TPC in the extracts of *Ocimum basilicum* and *Hyssopus officinalis* L. herbs cultivated in Romania (extraction with 70% ethanol on a waterbath) was 175.57 mg GAE/g and 77.72 mg GAE/g of raw material, respectively. The researchers revealed that TPC in the aerial parts of eight *Lamiaceae* species from Manipur (India) extracted with 80% ethanol varied in the range of 21.39–46.28 mg GAE/g of the herb (Khomdram and Potsangbam, 2011). The TPC in the *Marrubium peregrinum* L. herb collected in Serbia ranged from 27.26 to 89.78 mg GAE/g dependently of solvents (methanol, water, ethyl-acetate, acetone, or petroleum ether) used for maceration (Stanković, 2011). The TPC value of the 65–70% ethanol extracts of *Salvia sclarea* herb cultivated in Ukraine varied from 6.69 to 14.91 mg GAE per 1 gram of the herb (Hudz et al., 2019). The decoction of *Origanum vulgare* L. herb was characterized by the highest concentration of flavonoids and TPC than the infusion or hydroalcoholic extract (Martins et al., 2014). Thus, the mean TPC in the plant raw materials of the *Lamiaceae* representatives highly depends on the species of plant, its geographical origin, as well as solvents and extraction procedures chosen for analysis.

**FIGURE 1:**
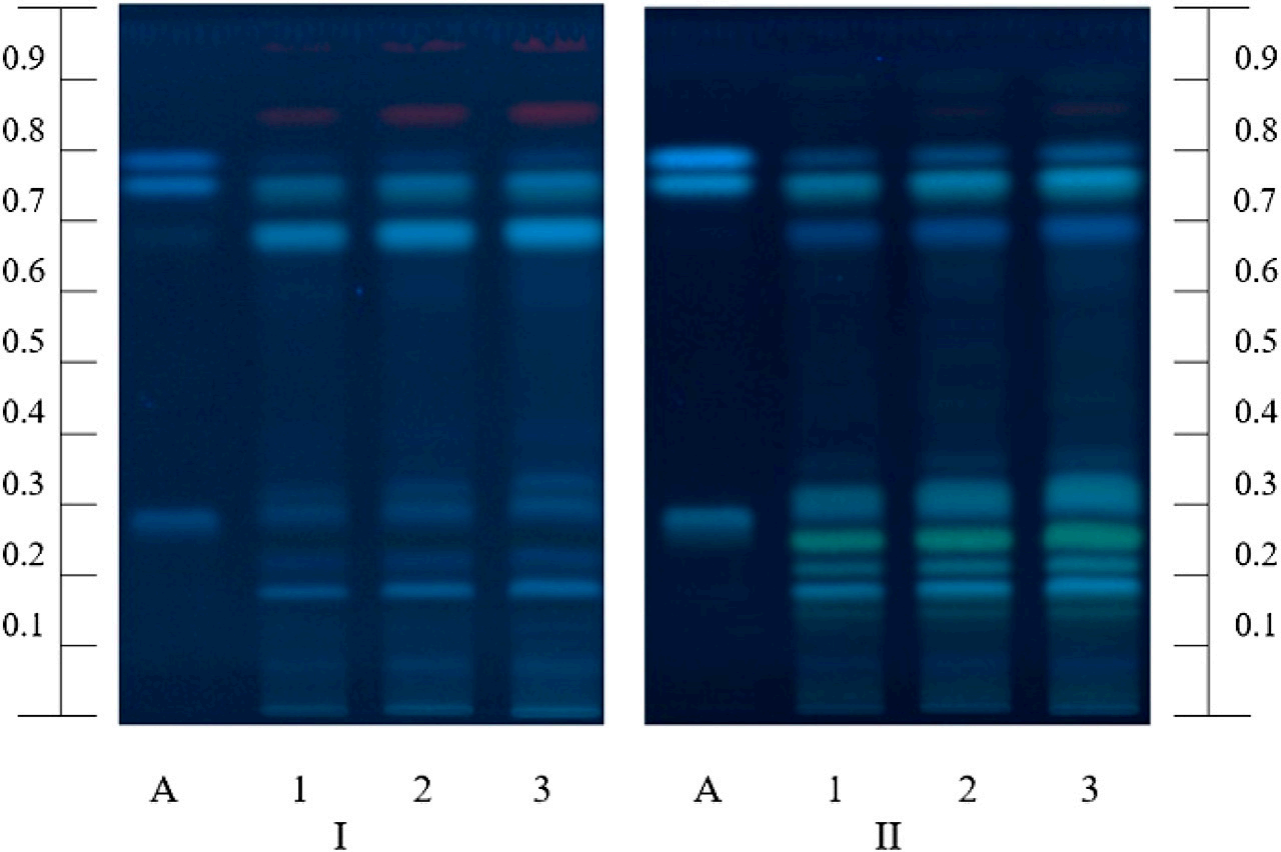
HPTLC fingerprints of the MFDE test solution (1–3) and polyphenol standards (A, chlorogenic, rosmarinic, and caffeic acids with increasing R_f_) at λ = 366 nm before (I) and after (II) derivatization.

The chromatographic fingerprints of polyphenols in the MFDE were obtained using the HPTLC method. The zones sequence presented in the chromatograms of the reference standards and test solution scanned at 366 nm before and after derivatization with 1% AlCl_3_ solution are given in Figure 1. The chromatograms of the test solution demonstrated the most intense light blue zones at R_*f*_ = 0.75 corresponding to rosmarinic acid; weaker light blue zones of caffeic acid were presented just above the rosmarinic acid spots (R_*f*_ = 0.79). Furthermore, several unidentified fluorescent zones in different shades of blue and yellow colors were visible in the chromatograms of the test solution, especially after the derivatization. These spots could be considered as hydroxycinnamic acids or flavonoids, respectively (Stanek et al., 2019; Shanaida et al., 2020). Similar results were found for the methanol extract of the *M. fistulosa* herb (Shanaida et al., 2020).

**FIGURE 2:**
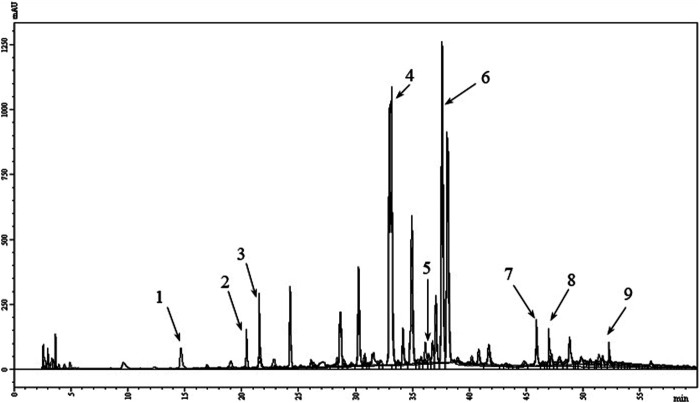
HPLC chromatogram of polyphenols in the MFDE. 1, neochlorogenic acid; 2, chlorogenic acid; 3, caffeic acid; 4, luteolin-7-*O*-glucoside; 5, apigenin-7-*O*-glucoside; 6, rosmarinic acid; 7, acacetin-7-*O*-glucoside; 8, luteolin; 9, apigenin.

The quantification of phenolic compounds in the MFDE was conducted using the HPLC method. The amounts of several components including hydroxycinnamic acids (rosmarinic, caffeic, chlorogenic, and neochlorogenic) and flavone derivatives (luteolin-7-*O*-glucoside, apigenin-7-*O*-glucoside, acacetin-7-*O*-glucoside, luteolin, and apigenin) were higher than 5 mg/g (Table 2; Figure 2).

**TABLE 2:** Contents of polyphenols in the MFDE evaluated by HPLC analysis.

Compound	Retention time (min)	Content (mg/g)
Neochlorogenic acid	14.8	5.62 ± 0.12
Chlorogenic acid	20.4	11.34 ± 0.14
Caffeic acid	21.6	21.62 ± 0.17
Luteolin-7-*O*-glucoside	33.1	76.30 ± 1.50
Apigenin-7-*O*-glucoside	36.8	6.91 ± 0.11
Rosmarinic acid	37.8	91.23 ± 1.62
Acacetin-7-*O*-glucoside	45.8	9.91 ± 0.15
Luteolin	47.0	9.54 ± 0.14
Apigenin	52.4	15.12 ± 0.15

**FIGURE 3:**
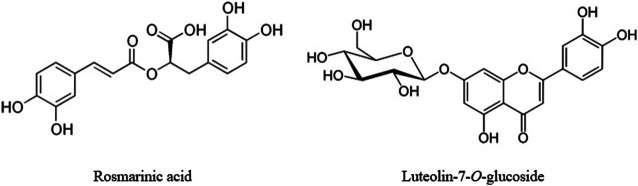
Structural formulas of the predominant polyphenols of the MFDE.

Rosmarinic acid (Figure 3), a major component of the MFDE, possesses significant antioxidant, antinociceptive, anti-inflammatory, hepatoprotective, immunomodulatory, antidiabetic, antiviral, and antimicrobial properties revealed in both *in vitro* and *in vivo* studies (Petersen and Simmonds, 2003; Rocha et al., 2015; Alagawany et al., 2017; Luo et al., 2020). Numerous data about the chemical profiles of the representatives of *Nepetoideae* subfamily of the *Lamiaceae* family demonstrated that rosmarinic acid is their common predominant phenolic compound (Lamien-Meda et al., 2010; Zhu et al., 2014; Benedec et al., 2015; Shanaida et al., 2018a; Jakovljević et al., 2020). Among several *Nepetoideae* species investigated by Benedec et al. (2015), the *Origanum vulgare* herb contained the maximum amount of rosmarinic acid (12.40 mg/g). The aerial part of *Satureja montana* L. accumulated up to 7.84 μg/mg of rosmarinic acid, depending on the applied technological parameters and solvents (Jakovljević et al., 2020). Rosmarinic acid, luteolin, and apigenin were the major polyphenols of 80% methanol extract obtained from the *Origanum vulgare* herb (Martins et al., 2014). Caffeic acid, which was found by the HPLC as one of the major components of the MFDE, possesses the noticeable antioxidant, anti-inflammatory, antiviral, and anticancer activities (Touaibia et al., 2011; Spagnol et al., 2019). Findings of Spagnol et al. (2019) demonstrated that the antioxidant activity of caffeic acid was greater than that of ascorbic acid, and it showed a higher level of stability than the last one.

The antioxidant and anti-inflammatory effects of flavones found in the MFDE were proven by many researchers (Aziz et al., 2018; Park and Song, 2013; Seyoum et al., 2006; Song and Park, 2014; Žemlička et al., 2014). Luteolin-7-*O*-glucoside (Figure 3) and its aglycon luteolin potently strengthen the HO-1-mediated antioxidative effect (Park and Song, 2013; Song and Park, 2014). Luteolin is characterized by the most likely orthopositions of OH-groups to donate the hydrogen atom (4′-OH and 3′-OH) (Seyoum et al., 2006). The significant free radical scavenging activity of luteolin-7-*O*-glucoside was confirmed using the ABTS assay (Žemlička et al., 2014). Aziz et al. (2018) pointed out that plants with a prominent content of luteolin have been used in a folk medicine to treat inflammation-associated diseases. Numerous *in silico, in vitro*, and *in vivo* experiments confirmed the strong anti-inflammatory activity of this flavone derivative (Aziz et al., 2018). Antioxidant properties of apigenin are also well known, and it is considered to be an effective therapeutic agent in the treatment of inflammation, neurodegenerative disease, and even cancer (Salehi et al., 2019).

The *in vitro* DPPH radical scavenging assay showed that the MFDE possesses strong antiradical activity with an IC_50_ value of 0.285 mg/mL. It is possible to compare the obtained results of DPPH inhibition with data of the other researchers of *Lamiaceae* species. Several *in vitro* assays revealed a higher antioxidant potential of water extracts from the *Nepeta* spp. postdistillation residues comparatively to acetone extracts isolated from its dried waste (Baranauskienė et al., 2019). The extracts from the *Ocimum basilicum* and *Hyssopus officinalis* herbs prepared with 70% ethanol showed the prominent DPPH free radical inhibiting activity with IC_50_ values of 0.1249 and 0.1254 mg/mL, respectively (Vlase et al., 2014). The methanol extract of *Marrubium peregrinum* herb demonstrated stronger antioxidant activity against DPPH radical (IC_50_ = 0.187 mg/mL) than its water extract (IC_50_ = 0.481 mg/mL) which indicates the positive effect of lower alcohols on the extraction of polyphenols from the plant raw material (Stanković, 2011). As the free radical scavenging effect depends on the number and location of OH-groups, the major components of MFDE such as rosmarinic acid with 4 hydroxyl groups and luteolin-7-*O*-glucoside containing three of them in the aglycon part possess the significant antioxidant potential (Cao et al., 2005; Zhu et al., 2014; Benedec et al., 2015). The distinct antiradical activity of rosmarinic acid is amplified by the orthoposition of hydroxyl groups on the rings A and B (Cao et al., 2005).

Polyphenols are regarded as the main antioxidants of plant raw materials of many *Lamiaceae* species (Dai and Mumper, 2010; Khomdram and Potsangbam, 2011; Vlase et al., 2014; Zhu et al., 2014; Asha et al., 2015; Benedec et al., 2015; Gougoulias and Masev, 2015; Tungmunnithum et al., 2018; Jakovljević et al., 2020). The healing effect of *Satureja montana* extract rich in caffeic, rosmarinic, and syringic acids and flavonoid rutin against cyclophosphamide-induced testicular damage in rats was due to its antioxidative and antiapoptotic mechanisms (El Tawab et al., 2014). *Origanum vulgare* extracts demonstrated the highest antioxidant capacity among the other *Lamiaceae* species that is in line with the high contents of rosmarinic acid and other polyphenols. A lot of phenolic compounds demonstrated the free radicals scavenging and anti-inflammatory effects that could have preventive or therapeutic effects for neurodegenerative and cardiovascular diseases and cancer (Cory et al., 2018; Rytsyk et al., 2020). Some polyphenols exert even higher antioxidant activity than vitamins (Brglez Mojzer et al., 2016; Spagnol et al., 2019).

### Biological Activities and Safety of MFDE

In the acute toxicity study, the general behavior of rats was recorded for 14 days after the MFDE administration. The results of this study indicate that the MFDE administered at doses of 500, 1,500, and 5,000 mg/kg did not induce any side effects or death of the experimental animals. No mortalities and other signs of adverse effects such as respiratory distress, convulsions, and changes of activity occurred in the animals tested during 14 days of behavioral observation. It suggests that up to 5,000 mg/kg the MFDE could be considered as safe.

Inflammation is a pathophysiological response of mammalian tissues to infections, injuries, allergies, or tumor growth. The administration of carrageenan as a high-molecular-weight polysaccharide into the animal paw leads to an inflammatory process as it is capable of releasing mediators associated with acute inflammation. It causes the time-dependent increase paw oedema (Luo et al., 2020). Nowadays, it is very important to develop effective herbal medicinal products with anti-inflammatory activity which possesses less side effects than synthetic drugs such as addiction and gastric ulcer.

The investigated MFDE significantly decreases in paw oedema of rats caused by the injection of carrageenan compared to the control group at 3 h of induced inflammation (Table 3). Anti-inflammatory activity was manifested by the MFDE in a dose-dependent manner; the highest tested dose (200 mg/kg) reduced paw oedema mostly (by 31.91% at the 3 h). The MFDE at a dose of 50 mg/kg did not demonstrate the noticeable anti-inflammatory effect when compared with the reference drug; this dose significantly inhibited paw oedema only after 3 and 6 h when compared with the control. The reference drug diclofenac caused a significant decrease paw oedema at all the hours of the experiment. According to different *in vitro* and *in vivo* studies (Petersen and Simmonds, 2003; Rocha et al., 2015; Luo et al., 2020), rosmarinic acid, which was found to be the predominant component of the MFDE, demonstrated the prominent anti-inflammatory effect due to the inhibition of lipoxygenase and cyclooxygenase pathways. Researchers revealed that administration of pure rosmarinic acid at a dose of 25 mg/kg reduced paw oedema in rats at 6 h by over 60% (Rocha et al., 2015). Such components of the MFDE as caffeic and chlorogenic acids (Touaibia et al., 2011; Naveed et al., 2018; Spagnol et al., 2019), as well as flavonoids such as apigenin, luteolin, and their glycosides (Xagorari et al., 2001; Aziz et al., 2018; Fan et al., 2018; Salehi et al., 2019), also possess the significant anti-inflammatory potential. Luteolin showed a significant anti-inflammatory activity in mice models induced by the carrageenan and reduced the number of abdominal constrictions caused by the acetic acid (Fan et al., 2018). Luteolin decreased the proinflammatory cytokine production in macrophages and endotoxin-stimulated phosphorylation cascade in murine (Xagorari et al., 2001). Thus, it can be speculated that the MFDE rich in rosmarinic acid and other polyphenols exhibits the anti-inflammatory activity via the inhibition of inflammatory cytokines production (Libby, 2007).

**TABLE 3:** Effect of the MFDE on carrageenan-induced paw oedema in rats.

Treatment	Dose (mg/kg)	Increase of paw oedema					
		After 1 h		After 3 h		After 6 h	
		Df	% AIA	Df	% AIA	Df	% AIA
Control	—	0.38 ± 0.02	—	0.47 ± 0.02	—	0.44 ± 0.02	—
MFDE	50	0.35 ± 0.02^b^	7.89	0.38 ± 0.02^a^ ^,^ ^b^	19.15	0.36 ± 0.02^a^ ^,^ ^b^	18.18
MFDE	100	0.31 ± 0.01^a^ ^,^ ^b^	18.42	0.34 ± 0.02^a^	27.66	0.35 ± 0.02^a^	20.95
MFDE	200	0.30 ± 0.01^a^ ^,^ ^b^	21.05	0.32 ± 0.01^a^	31.91	0.34 ± 0.01^a^	22.72
Diclofenac	8	0.22 ± 0.01^a^	42.1	0.25 ± 0.02^a^	46.87	0.34 ± 0.02^a^	22.72

Df, difference in paw volume before and after carrageenan injection.

^a^ Significantly different compared to untreated control (*р* ≤ 0.05).

^b^ Significantly different compared to the diclofenac group (*р* ≤ 0.05). Values are expressed as mean ± SEM of six individual values.

The writhing test induced by acetic acid is a widespread model to evaluate the peripheral antinociceptive effects of drugs (Karim et al., 2019). The pain provoked by the injection of acetic acid intraperitoneally is the consequence of irritation of chemosensitive nociceptors and causes releasing algogenic compounds such as prostaglandins, serotonin, histamine, and bradykinin (Magne et al., 2016). Table 4 shows that the analgesic effect the MFDE is manifested in a dose-dependent manner. The animals of the control group demonstrated 63.27 ± 1.49 writhing count, while the MFDE at a dose of 50 mg/kg reduced it to 49.8 ± 1.21. Higher doses of the extract decreased the writhing counts more significantly: 100 mg/kg to 36.24 ± 0.76 and 200 mg/kg to 30.85 ± 0.71. Thus, the MFDE in the highest dose (200 mg/kg) induced a noticeable reduction in the number of writhing in the mice provoked by the injection of acetic acid which was close to the reference drug. The revealed pharmacological effect of the MFDE could be attributed to a significant amount of polyphenols. A similar analgesic effect was found for the high dose of aqueous extract of *Plectranthus glandulosus* Hook. (*Lamiaceae*) leaves rich in phenolic compounds (Magne et al., 2016). The aqueous extract from the *Rosmarinus officinalis* L. leaves in a dose range of 100–400 mg/kg and rosmarinic acid at the doses of 10–40 mg/kg demonstrated their effectiveness in the management of pain and inflammation (Lucarini et al., 2013). The healing properties of rosmarinic acid were found in the experimental neuropathic pain in rats, and its anti-inflammatory and antiapoptotic activities have the key role in the observed antinociceptive effect (Rahbardar et al., 2018). *In vitro* and *in vivo* studies of the extracts prepared from the aerial parts of a lot of the *Lamiaceae* species from genera such as *Mentha* L.*, Ocimum* L.*, Salvia* L.*, Melissa* L., *Satureja* L.*,* and *Rosmarinus* L. showed their potent analgesic activity, and the revealed therapeutic effect could be attributed to the high polyphenol levels (Lucarini et al., 2013; Uritu et al., 2018).

**TABLE 4:** Effect of the MFDE on pain induced by acetic acid in mice.

Treatment	Dose (mg/kg)	No. of writhings	Inhibition (%)
Control	—	63.27 ± 1.49	—
MFDE	50	49.8 ± 1.21^a^ ^,^ ^b^	21.29
MFDE	100	36.24 ± 0.76^a^ ^,^ ^b^	42.72
MFDE	200	30.85 ± 0.71^a^	51.24
Metamizole sodium	55	28.67 ± 0.68^a^	54.68

^a^ Significantly different compared to untreated control (*р* ≤ 0.05).

^b^ Significantly different compared to the analgin group (*р* ≤ 0.05). Values are expressed as mean ± SEM of six individual values.

## Conclusion

This is the first research of the polyphenol profiles and pharmacological activities of the hydrodistilled residue by-product obtained from the *M. fistulosa* herb. The complex processing of the *M. fistulosa* raw material allows using rationally its post-hydrodistillation waste simultaneously with obtaining essential oil that enhances the overall profitability of this aromatic plant. The chromatographic analyses of polyphenols in the obtained MFDE revealed a high amount of rosmarinic acid, luteolin-7-*O*-glucoside, and other polyphenols with the valuable therapeutic properties. The high TPC in the MFDE correlated with its potent IC_50_ value. The administration of the obtained extract at the doses up to 5,000 mg/kg did not induce any toxic reactions. The *in vivo* assay revealed dose-dependent anti-inflammatory and analgesic activities of MFDE. Therefore, the results of this study indicate that hydrodistilled residue by-product from the *M. fistulosa* herb may represent a new natural source of polyphenols with significant pharmacological potential. The investigated extract could be also exploited as a health-promoting substance in food or cosmetic products.

## Data Availability

The raw data supporting the conclusions of this article will be made available by the authors, without undue reservation, to any qualified researcher.
